# Co-dosing Ozone and Deionized Water as Oxidant Precursors of ZnO Thin Film Growth by Atomic Layer Deposition

**DOI:** 10.1186/s11671-020-03382-1

**Published:** 2020-07-29

**Authors:** Yung-Chen Cheng, Hsiang-Chen Wang, Shih-Wei Feng, Tsai-Pei Li, Siu-Keung Fung, Kai-Yun Yuan, Miin-Jang Chen

**Affiliations:** 1 Department of Materials Science, National University of Tainan, Tainan, 70005 Taiwan; 2 Department of Mechanical Engineering and Advanced Institute of Manufacturing with High-tech Innovations, National Chung Cheng University, Chia Yi, 62102 Taiwan; 3 Department of Applied Physics, National University of Kaohsiung, Kaohsiung, 81148 Taiwan; 4 Department of Materials Science and Engineering, National Taiwan University, Taipei, 10617 Taiwan

**Keywords:** Zinc oxide, Atomic layer deposition, Strain relaxation, Ozone precursor, Thermal annealing, Photoluminescence

## Abstract

Characteristics of atomic layer deposition (ALD)-grown ZnO thin films on sapphire substrates with and without three-pulsed ozone (O_3_) as oxidant precursor and post-deposition thermal annealing (TA) are investigated. Deposition temperature and thickness of ZnO epilayers are 180 °C and 85 nm, respectively. Post-deposition thermal annealing is conducted at 300 °C in the ambience of oxygen (O_2_) for 1 h. With strong oxidizing agent O_3_ and post-deposition TA in growing ZnO, intrinsic strain and stress are reduced to 0.49% and 2.22 GPa, respectively, with extremely low background electron concentration (9.4 × 10^15^ cm^−3^). This is originated from a lower density of thermally activated defects in the analyses of thermal quenching of the integrated intensity of photoluminescence (PL) spectra. TA further facilitates recrystallization forming more defect-free grains and then reduces strain and stress state causing a remarkable decrease of electron concentration and melioration of surface roughness.

## Introduction

Several oxidizing agents are used in the growth of ZnO. They include water (H_2_O), hydrogen peroxide (H_2_O_2_), oxygen (O_2_), and ozone (O_3_) [1–6]. H_2_O is a commonly used oxidant in the growth of ZnO with atomic layer deposition (ALD). ALD is a layer-by-layer self-limiting growth mechanism. Specific surface ligands exchange reactions with sequential pulsing of respective precursors. Surface reactions stop and saturate when the reactive sites of the surface are completely depleted. ALD growth of thin films has advantages such as superior conformal deposition on surfaces and side regions, excellent step coverages of edges, high uniformity over a large area, precision in layer thickness control, and suitable for low deposition temperature [7–9].

Strong oxidizing agents influence not only the material structures but optoelectrical characteristics of ALD-deposited ZnO. H_2_O_2_ oxidant provides more oxygen-rich conditions than commonly used H_2_O precursor to passivate defects oxygen vacancies (V_O_) and zinc interstitials (Zn_*i*_) in ALD-grown ZnO films at low growth temperatures from 80 to 150 °C. Columnar surface morphologies with (002) preferential orientation of growth plane occurred when the use of oxidant is altered from H_2_O to H_2_O_2_ [2]. H_2_O_2_ oxidant increases the growth rates by approximately 70% as compared with using O_3_ gas reactant of ALD-grown ZnO at 200 °C. Rise of hydroxyl (OH) group density on the growing surface of the films is responsible for the increase of growth rates [3]. The increase of growth rate of ZnO about 60% for using pure O_2_ instead of H_2_O as an oxidizer is reported, too [4].

O_3_ is an effective oxidizer in ALD-grown oxide materials. The high electrochemical potential of O_3_ gives rise to fast reaction rates at a low growth temperature. O_3_ is more volatile than H_2_O, H_2_O_2_, and O_2_, and it is easier to purge. Therefore, purge times in each cycle can be shortened. The absence of hydrogen in O_3_ molecule leads to less hydrogen and hydroxyl contamination in the growth. Less uniform in thickness due to recombinative surface loss of ZnO is shown for 10 s of O_3_ exposure times when the growth is at 200 °C [5]. ALD-prepared undoped ZnO films with O_3_ oxidizing agents show a double thermoelectric power factor compared to samples with H_2_O oxidizer. H_2_O- and O_3_-grown ZnO films have the same defect levels of V_O_ but different Zn-related defect levels. Sufficient oxidation power of the O_3_ results in a lower content of native Zn_*i*_ and hence a larger thermoelectric power factor. Strong oxidant effect of O_3_ elevates the thermoelectric performance of undoped ZnO films [6]. Co-dosing O_3_ and H_2_O could improve the uniformity and conformality of ZnO thin film for proper ALD processes [5].

Intrinsic and extrinsic strains exist in ZnO film grown on a sapphire substrate naturally. Intrinsic strain is originated from high-density crystallographic imperfections in ZnO. Crystallographic imperfections include hydrogen complexes, zinc interstitials (Zn_*i*_), oxygen vacancies (V_O_), threading dislocations (TDs), and grain boundaries (GBs) [10–21]. Extrinsic strain is generated from a large mismatch of lattice constants and thermal expansion coefficients between ZnO epilayer and sapphire substrate. Various manners are exerted to diminish the intrinsic and extrinsic strain of ZnO materials on sapphires. Thin MgO buffer layer can diminish extrinsic strain, reduce surface roughness by 58.8%, and suppress surface cracks of ZnO thin film on sapphire [22]. Extrinsic strain of ZnO on sapphire is fully relaxed with the thickness reaching 30 nm prepared by magnetron sputter deposition at 550 °C [23]. Relaxation of compressive stress from 1.77 to 1.47 GPa of ZnO films deposited by spray pyrolysis method with the rise of glass substrate temperatures from 350 to 450 °C is exhibited [24].

ALD ZnO grown at 180 °C with one-pulsed precursors (DEZn and H_2_O) shows background electron concentration as high as ~ 10^18^ cm^−3^ even with post-deposition TA [25]. Commonly used one-pulsed H_2_O precursors of ALD processes do not generate ideal monolayer of oxygen atoms. Three-pulsed precursors (DEZn and H_2_O) could generate multiple hits or collisions of precursor molecules with surface ligand to promote the reaction probability for the ALD ZnO grown at low temperature 100 °C. The choice of “three” pulses helps securing of reactants locating the open chemisorption or reaction site properly. Extremely low background electron concentration 8.4 × 10^14^ cm^−3^, high electron drift mobility 62.1 cm^2^/Vs, and pronounced enhancement of near bandgap edge (NBE) photoluminescence (PL) of three-pulsed precursors ZnO with suitable buffer layer and RTA conditions are acquired [26]. Several reports show enhancement of material quality of ZnO thin films and ZnO/ZnMgO multiple quantum wells with thermal annealing [27, 28]. In this report, three-pulsed O_3_ and subsequent one-pulsed H_2_O as oxidizers per ALD cycle are used to grow ZnO thin films at 180 °C. Post-deposition thermal annealing (TA) is applied to improve crystalline quality of the samples. Material micro- and nano-structural, photoluminescence, and Hall effect features of ALD-grown ZnO thin films are explored.

## Experimental Methods

**Fig. 1:**
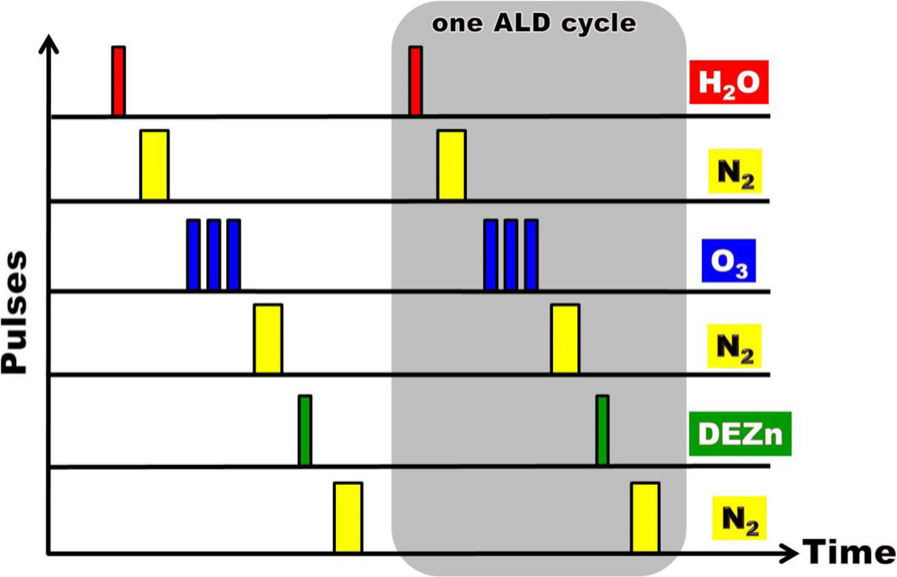
ALD-grown ZnO films on sapphire substrates with pulse sequence H_2_O/N_2_/O_3_/N_2_/DEZn/N_2_ using DI water, O_3_, and Zn(C_2_H_5_)_2_ as the precursors

ZnO epilayers are deposited on conventional c-face sapphire (c-Al_2_O_3_) substrates by Cambridge NanoTech Savannah 100 ALD system. In the growth of ALD ZnO thin films, precursors including deionized (DI) H_2_O, O_3_, and diethyl zinc (DEZn, Zn(C_2_H_5_)_2_) are utilized. Table 1 demonstrates conditions of pulse number of O_3_ and post-deposition TA of three specimens named A, B, and C. A schematic diagram of pulse sequence with time per ALD cycle of samples is displayed in Fig. 1. In this figure, one ALD cycle contains six sequential steps. The first step is the injection of one-pulsed deionized (DI) H_2_O into a meter-scale reactor to make hydroxyl (OH)-terminated surface on sapphire or to react with the dangling ethyl groups (C_2_H_5_) forming zinc-oxygen (Zn–O) bridges on Zn surface with hydroxyl surface groups. One atomic layer of oxygen (O) is produced. The second step is the purge of high purity nitrogen gas (N_2_) to remove excess precursor molecules and volatile byproducts and to prevent mixing of subsequent precursors after each exposure of reactants. The third step is the injection of three-pulsed O_3_ into reactors to facilitate the reduction of native defects. The fourth step is the purge of the chamber by N_2_ gas. The fifth step is the injection of one-pulsed DEZn into reactors to produce one atomic layer of zinc (Zn) upon the oxygen layer. The final step is also the purge of the chamber by N_2_ gas. Precursors pulsed into the reaction chamber through carry gas N_2_ with a chamber pressure of 4 × 10^−1^ Torr. The optimal condition of exposure time for the reactants DI H_2_O, O_3_, and DEZn is 0.01, 0.5, and 0.015 s, respectively. The pulse time of the evacuation of the chamber is 5 s. The thickness of ZnO thin films is 85 nm with 500 ALD cycles of each sample. Other favorable conditions of deposition parameters are shown in the previous reports [29]. Post-deposition TA at 300 °C in the ambience of O_2_ for 1 h in a furnace is processed on sample C.

**Table 1:** Pulse number of ozone, thermal annealing, strain, and stress of specimens

Sample	Pulse number of ozone	Thermal annealing	Strain (%)	Stress (GPa)
A	0	No	1.08	4.90
B	3	No	0.74	3.37
C	3	300 °C, 1 h, O_2_	0.49	2.22

Material structural, optical, and electrical properties of samples are conducted by the following measurements. The X-ray diffraction (XRD) patterns are measured with diffraction angle (2*θ*) range from 33 to 38° and wavelength 0.154 nm of copper Kα radiation by using the instrument D2 phaser (Bruker Corporation). Hall effect measurement uses Ecopia HMS-3000 system, and specimens are cut into squares having a size area of 0.7 × 0.7 cm^2^. Four corners of the specimens are soldered by small indium blobs showing ohmic contacts in van der Pauw configuration. Sheet carrier density, mobility, and resistivity of the films are measured. Photoluminescence (PL) spectra are carried out from 10 to 300 K with an excitation wavelength of 325 nm and a power of 55 mW of He–Cd laser. Thermal quenching of integrated intensity of PL spectra with the increase of temperature is analyzed. Surface texture and roughness of specimens examined from high-resolution images of atomic force microscope (AFM) are taken by the instrument of Veeco Dimension 3100.

## Results and Discussion

**Fig. 2:**
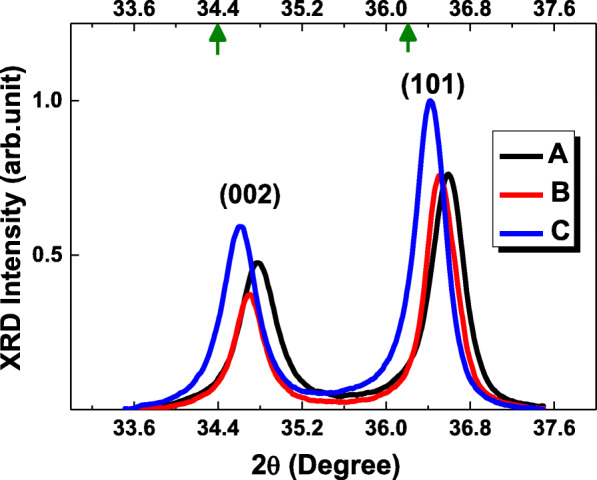
XRD patterns of ZnO films with crystalline orientations along (002) and (101) directions of the specimens. The arrows indicate the diffraction angles 34.4° and 36.2° of (002) and (101) of bulk ZnO, respectively

Figure 2 demonstrates the crystalline nature of the specimens by the measurements of XRD patterns. Two Bragg diffraction peaks (002) and (101) correspond to the hexagonal wurtzite structure of ZnO. The strongest peak intensity of XRD patterns is normalized for comparison of peak intensity among samples. Two green arrows indicated on the top horizontal axis show diffraction angles 34.4° and 36.2° of (002) and (101), respectively, of strain-free bulk ZnO acquired from the material data sets released by the organization of Joint Committee on Powder Diffraction Standards (JCPDS). In the figure, one can observe the (002) and (101) peaks in sample B and C approach and further approach diffraction angles of (002) and (101) of strain-free bulk ZnO.

Biaxial strain along the *c*-axis of the epitaxial layers can be obtained through the shift of XRD patterns along (002) in contrast with strain-free bulk ZnO. Average strain (*ε*_*z*_) in the lattice of ZnO films is estimated from the lattice parameters using the following expression.

1 $$ {\varepsilon}_z=\frac{c-{c}_0}{c_0}\times 100\% $$

where *c* and *c*_0_ are the lattice constants along the *c*-axis calculated from Bragg’s diffraction angles of (002) peaks of ZnO films and bulk. The average stress (*σ*) in the plane of the films can be calculated using the biaxial strain model:

$\sigma =\frac{2{c}_{13}^2-{c}_{33}\left({c}_{11}+{c}_{12}\right)}{c_{13}}\times {\varepsilon}_z=-453.6\times {\varepsilon}_z\left(\mathrm{GPa}\right)$   (2)where *c*_11_ = 209.7 GPa, *c*_12_ = 121.1 GPa, *c*_13_ = 105.1 GPa, and *c*_33_ = 210.9 GPa are the elastic stiffness constants of bulk ZnO. In Table 1, the strains/stresses (*ε*_*z*_/*σ*) of A, B, and C samples are 1.08%/4.90 GPa, 0.74%/3.37 GPa, and 0.49%/2.22 GPa, respectively. Strain/stress is reduced and further reduced in samples B and C.

**Fig. 3:**
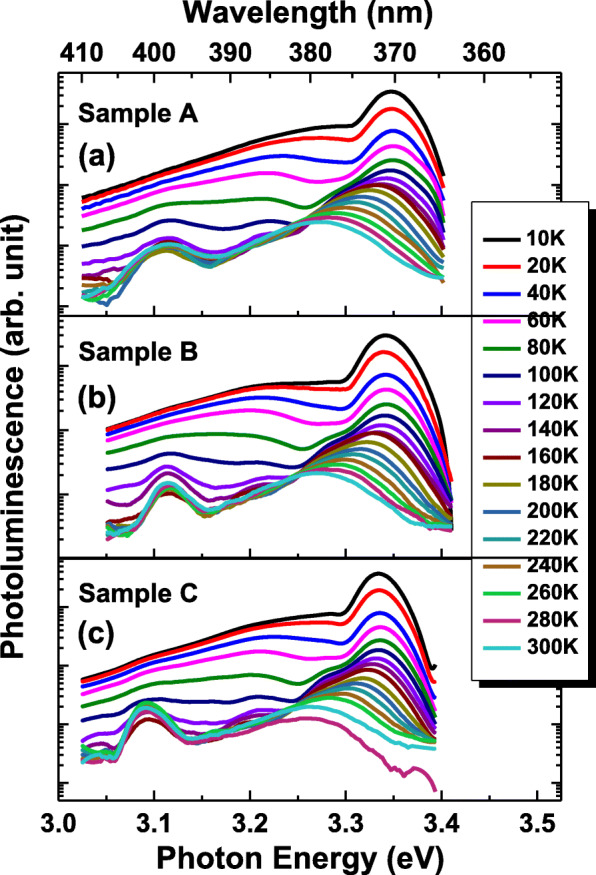
**a**–**c** Temperature-dependent PL spectra of specimens

**Fig. 4:**
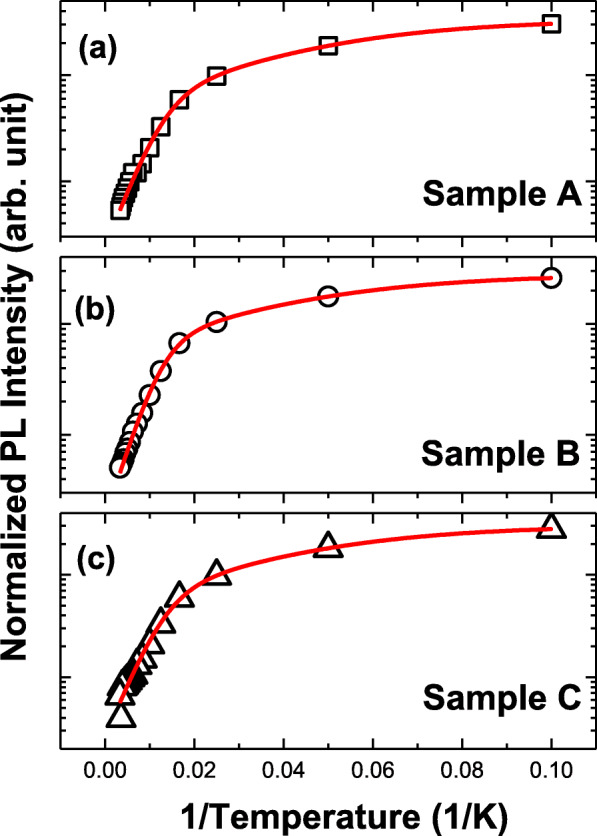
**a**–**c** Arrhenius plots of thermal quenching of integrated intensity of PL spectra and red fitting curves of samples

PL spectra at various temperatures from 10 to 300 K of samples are displayed in Fig. 3. Strong near band edge radiative recombination of excitons with spectral peak energy around 3.34 eV is dominant in PL spectra of all samples. Longitudinal-optical (LO) phonon-assisted optical emission is observed at the lower energy shoulder of the PL spectra of samples. In Fig. 4a–c, it exhibits the Arrhenius plot of integrated intensity of PL spectra versus the inverse of temperature. Thermal quenching of integrated intensity of PL with increasing temperature can be fitted by the following Arrhenius formula.

3 $$ I(T)=\frac{A}{1+{D}_{nr1}\exp \left(\frac{-{E}_{A1}}{k_{\mathrm{B}}T}\right)+{D}_{nr2}\exp \left(\frac{-{E}_{A2}}{k_{\mathrm{B}}T}\right)} $$

where *I*(*T*) represents the integrated PL intensity. *A* is a constant. *D*_*nr*1_ and *D*_*nr*2_ are constants related to the density of non-radiative recombination centers. *E*_*A*1_ and *E*_*A*2_ are the activation energies corresponding to the non-radiative recombination process of donor bound excitons at low temperature and free excitons at high temperature, respectively. *k*_B_ is the Boltzmann constant. Least squares method in regression analysis is utilized to fit the data showing the parameters of *D*_*nr*1_, *D*_*nr*2_, *E*_*A*1_, and *E*_*A*2_ with red fitting curves in Table 2 and Fig. 4a–c. The fitting result shows that variations of *D*_*nr*1_, *E*_*A*1_, and *E*_*A*2_ among samples are light. *D*_*nr*2_ are 132.7, 153.6, and 110.8 of samples A, B, and C, respectively, showing a large difference in the amount of defect density. The smallest value of *D*_*nr*2_ suggests the lowest density of thermally activated defects in sample C.

**Table 2:** Carrier concentration, mobility, and resistivity of Hall effect measurements and root mean square (rms) surface roughness estimated by AFM images of specimens

Sample	Carrier concentration (cm^−3^)	Mobility (cm^2^/Vs)	Resistivity (Ωcm)	*D*_*nr*1_	*E*_*A*1_ (meV)	*D*_*nr*2_	*E*_*A*2_ (meV)	Roughness (nm)
A	3.4 × 10^19^	19	9.7 × 10^−3^	6.26	3.6	132.7	22.3	1.92
B	4.6 × 10^17^	1.1	1.2 × 10^1^	4.3	3.4	153.6	25.6	4.30
C	9.4 × 10^15^	6.1	1.1 × 10^2^	5.29	3.5	110.8	21.7	2.18

Background electron concentration, mobility, and resistivity of Hall effect measurements of samples are listed in Table 2. In sample B, two orders of magnitude decrease of carrier concentration with reduction of mobility are shown as compared with sample A. Further immense decrease of carrier concentration to the lowest value of 9.4 × 10^15^ cm^−3^ and the rise of mobility to a value 6.1 cm^2^/Vs are observed in sample C as compared with sample B. The least amount of electron concentration is due to the largest relaxation of strain/stress states and remarkable reduction of native defect density in sample C.

**Fig. 5:**
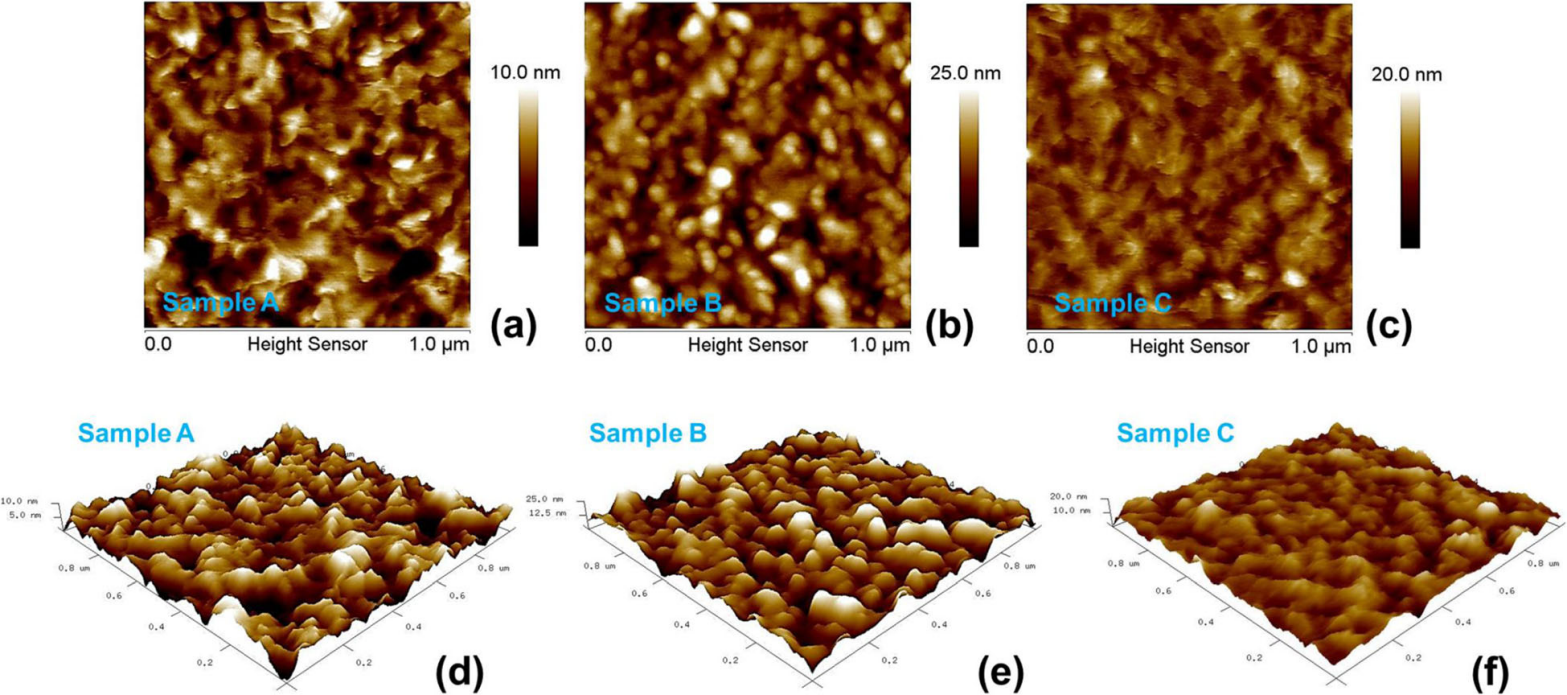
**a**–**c** 2D and **d**–**f** 3D AFM images of specimens. The scale of height in 2D images are presented on the right color bar

**Fig. 6:**
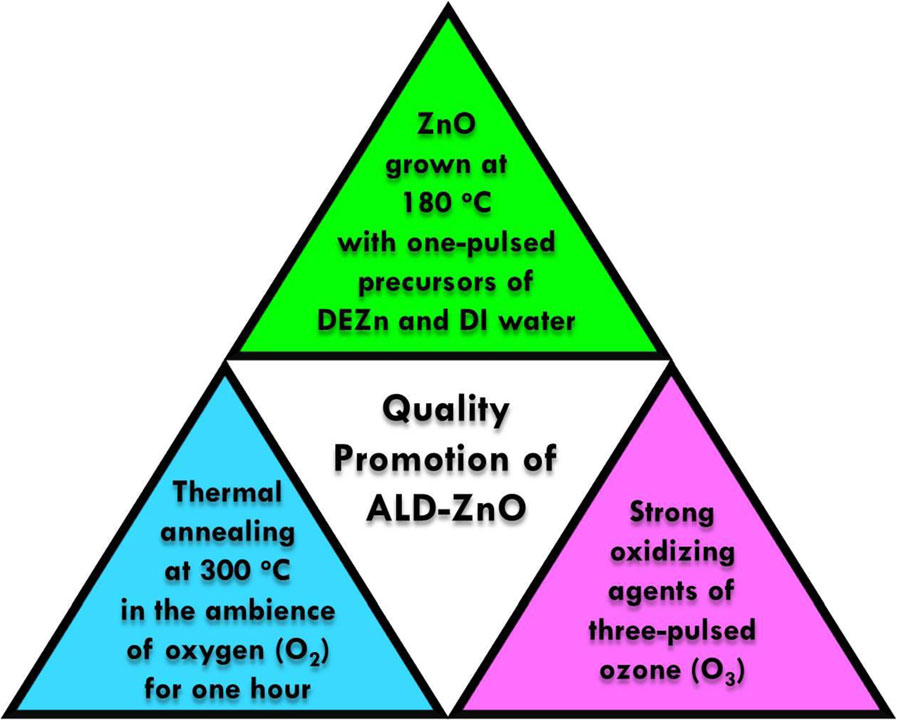
A pyramid diagram. In the pyramid, four triangles are illustrated in different colors. Three triangles at the edges of the pyramid show key growth and processing conditions of ZnO. Combination of these three crucial components in the growth promotes quality of ALD ZnO epilayers shown by the triangle at the center of the pyramid

Figures 5a–c and d–f are 2D and 3D AFM images of specimens. Root mean square (RMS) roughness of A, B, and C is 1.92, 4.30, and 2.18 nm, respectively, as shown in Table 2. The lowest surface texture roughness occurred in sample A. With the use of O_3_ precursor in sample B, surface roughness is increased. Reduction of spatial uniformity of the ALD ZnO films is due to the surface loss of O_3_ [21]. Surface loss of O_3_ is related to the transition from reaction- to recombination-limited growth and can constitute the primary atomic loss channel to destroy the films resulting in poor thickness uniformity. This is correlated to the reduction of diffraction peak intensity along (002) in sample B in Fig. 2. With the treatment of post-deposition TA in sample C, surface uniformity is meliorated. Meanwhile, a dramatic decrease of background electron concentration and increase of mobility are achieved. Thermal annealing brings about migration in crystal lattice; thus, metallurgic recrystallization takes place. Recrystallization accompanies the reduction in strength of strain/stress and intrinsic crystal lattice imperfections; therefore, better quality of ZnO thin film is attained. This result is consistent with the enhancement of two diffraction peak intensities in XRD pattern in sample C. It is worthy to note that electrons’ mobility can be affected by scattering sources such as impurities, lattices, and defects. That scattering sources could alter the average electron velocity. In general, reduction of defect density and hence decrease of electron concentration lead to the rise of mobility. In this report, an increase of roughness of surface texture due to the action of dosing ozone precursor could cause lower electron mobility in sample C than A. In Fig. 6, a pyramid diagram containing three triangles in different colors illustrates the three key growing and processing conditions to achieve high-quality ALD ZnO epilayers in this report.

## Conclusions

One-pulsed H_2_O and three-pulsed strong oxidant O_3_ precursors in the ALD processes can diminish the strain/stress and hence conspicuously reduce electron concentration in ZnO thin film but increase surface roughness. Post-deposition TA at 300 °C in the ambience of oxygen (O_2_) for 1 h can further facilitate the formation of more defect-free grains with lower strain/stress, lower background electron concentration, and melioration of surface roughness after the growth of using three-pulsed O_3_ precursors. The lowest strain/stress and background electron concentration which are 0.49%/2.22 GPa and 9.4 × 10^15^ cm^−3^, respectively, owing to the dramatic decrease of intrinsic native defect of ALD-grown ZnO thin films are attained.

## Data Availability

The data that support the findings of this study are available from the corresponding author (Yung-Chen Cheng) upon reasonable request.

## Abbreviations

ALD: Atomic layer deposition
O_3_: Ozone
TA: Thermal annealing
O_2_: Oxygen
PL: Photoluminescence
H_2_O: Water
H_2_O_2_: Hydrogen peroxide
OH: Hydroxyl
Zn_*i*_: Zinc interstitials
V_O_: Oxygen vacancies
TDs: Threading dislocations
GBs: Grain boundaries
NBE: Near bandgap edge
c-Al_2_O_3_: c-face sapphire
DI: Deionized
C_2_H_5_: Ethyl groups
N_2_: Nitrogen gas
Zn: Zinc
XRD: X-ray diffraction
AFM: Atomic force microscope
RMS: Root mean square
